# Integrated Multi-Omic Analyses of the Genomic Modifications by Gut Microbiome-Derived Metabolites of Epicatechin, 5-(4′-Hydroxyphenyl)-γ-Valerolactone, in TNFalpha-Stimulated Primary Human Brain Microvascular Endothelial Cells

**DOI:** 10.3389/fnins.2021.622640

**Published:** 2021-03-26

**Authors:** Karla Fabiola Corral-Jara, Saivageethi Nuthikattu, John Rutledge, Amparo Villablanca, Christine Morand, Hagen Schroeter, Dragan Milenkovic

**Affiliations:** ^1^INRAE, UNH, Université Clermont Auvergne, St Genes Champanelle, France; ^2^Division of Cardiovascular Medicine, University of California, Davis, Davis, CA, United States; ^3^Mars, Inc., McLean, VA, United States

**Keywords:** valerolactones, epicatechin, genomics, multi-omics, lncRNA, brain endothelial cells, systems biology, nutrigenomics

## Abstract

Cerebral blood vessels are lined with endothelial cells and form the blood-brain barrier. Their dysfunction constitutes a crucial event in the physiopathology of neurodegenerative disorders and cognitive impairment. Epicatechin can improve cognitive functions and lower the risk for Alzheimer’s disease or stroke. However, molecular mechanisms of epicatechin on brain vascular endothelium are still unexplored. The objective of this study was to investigate the biological effects of gut microbiome-derived metabolites of epicatechin, 5-(4′-Hydroxyphenyl)-γ-valerolactone-3′-sulfate and 5-(4′-Hydroxyphenyl)-γ-valerolactone-3′-O-glucuronide, in TNF-α-stimulated human brain microvascular endothelial cells at low (nM) concentrations by evaluating their multi-omic modification (expression of mRNA, microRNA, long non-coding RNAs, and proteins). We observed that metabolites are biologically active and can simultaneously modulate the expression of protein-coding and non-coding genes as well as proteins. Integrative bioinformatics analysis of obtained data revealed complex networks of genomics modifications by acting at different levels of regulation. Metabolites modulate cellular pathways including cell adhesion, cytoskeleton organization, focal adhesion, signaling pathways, pathways regulating endothelial permeability, and interaction with immune cells. This study demonstrates multimodal mechanisms of action by which epicatechin metabolites could preserve brain vascular endothelial cell integrity, presenting mechanisms of action underlying epicatechin neuroprotective properties.

## Introduction

Polyphenols are among the most abundant phytochemicals found in plant foods. They comprehend several families of compounds, with the most represented in the human diet being phenolic acids and flavonoids (Vogiatzoglou et al., 2015). Flavanols are a class of flavonoids found in fruits and they exist in monomeric (cathechins and epicatechins), and polymeric (proanthocyanidines) forms. They are commonly found in cocoa, tea, and various fruits, such as apple or grape. These flavanols play a key role in the beneficial health effects of fruits and vegetables as well as their derivatives (Crozier et al., 2009).

Numerous studies have shown that epicatechin is highly metabolized following its consumption. Epicatechin can be absorbed in the small intestine, rapidly conjugated by phase I and phase II detoxification enzymes, and appears in the circulation between 1–4 h after ingestion with major metabolites such as E3′G, E3′S, 3′ME5S, and 3′ME7S (Ottaviani et al., 2016). These metabolites are absent from plasma after 8 h, at the point when the ingested epicatechin reaches the intestine and the colon. In the colon, the microbiota can open the C-ring, resulting in the formation of 5-carbon side chain ring fission metabolites that can be further metabolized by the phase II metabolism by enzymes present in the colon and/or the liver resulting in the sulfated and glucuronidated forms of γ-valerolactones that can be detected in plasma. Two major gut metabolites derived catabolites that were identified in plasma are 5-(4′-Hydroxyphenyl)-γ-valerolactone-3′-sulfate and 5-(4′-Hydroxyphenyl)-γ-valerolactone-3′-O-glucuronide, at 272 ± 56 nM and 125 ± 30 nM, respectively (Ottaviani et al., 2016) and can remain in the blood stream for over 12 h. Very few studies suggested that these γ-valerolactones are bioactive by exerting anti-inflammatory properties or decreasing blood pressure (Mena et al., 2019), however, such studies remain very scarce.

In the context of health, flavonoids are of particular importance because *in vitro*, pre-clinical studies and randomized controlled trials (RCT) have shown that the flavanols exert positive effects on different cardiovascular disease risk factors, including blood pressure, vasodilation, or vascular stiffness (Schroeter et al., 2006; Desch et al., 2010). Hypertension and arterial stiffness are also the main risk factors for cerebrovascular injury. The role of cerebrovascular dysfunction in cognitive impairment is increasingly recognized (Nation et al., 2019). Its dysfunction can lead to accelerated brain atrophy, reduced cognitive ability, an increased risk of stroke, and an increased risk of neurodegenerative diseases, such as Alzheimer’s disease (AD), and dementia. It has been shown that aging can induce an increase in cerebral blood flow activated by neural activation, also known as neurovascular coupling, which is functional damage of cerebral microvessels and astrocytes (Tarantini et al., 2017). This can contribute to neurovascular dysfunction and consequently to cognitive decline with aging and induce the development of age-related neurodegenerative diseases (Tarantini et al., 2017).

Several studies suggest that epicatechin can improve different aspects of cognitive function in animals and humans. Flavonoids can maintain cognitive aptitudes during aging in rats, decrease the risk for developing AD, decrease the risk of stroke in humans, and can exert beneficial effects on cerebral blood flow (Nehlig, 2013; Wang et al., 2014). Health effects of consumption of these bioactive compounds on neurocognition and behavior, including age- and disease-related cognitive decline, were shown in animal models of aging, stroke, and dementia (Sokolov et al., 2013). A recent systematic review has suggested a positive effect of cocoa flavanols on executive function and memory (Barrera-Reyes et al., 2020). It has also been suggested that consumption of cocoa flavanol may improve regional cerebral perfusion (Lamport et al., 2015) and can also enhance dentate gyrus function and improves cognition in older adults (Brickman et al., 2014).

The cerebral blood vessels are lined with endothelial cells (EC) and connected by tight junctions. Together with astrocytes, microglia cells, pericytes, and the basement membrane, they form the blood-brain barrier (BBB) and represent an interactive cellular complex that regulates the entry of blood products, pathogens, and cells into the brain, which is essential for normal neuronal functioning, thus playing an important role in the protection and homeostasis of the brain. Dysfunction of the BBB also plays a major role in most neurodegenerative disorders, resulting in increased permeability of EC, which results in neuroinflammation that contributes to the neurodegeneration process (Palmer, 2011). BBB degradation is also an initial step in the aging human brain that starts in the hippocampus and can contribute to cognitive impairment (Montagne et al., 2015). It has therefore been suggested that the degradation of BBB is a sensitive and early measure of cognitive dysfunction in Alzheimer’s, Parkinson’s, and even multiple sclerosis (Sweeney et al., 2018; Nation et al., 2019). We have previously described that epicatechin metabolites can prevent endothelial dysfunction by reducing interactions between monocytes and TNF-α-stimulated vascular EC (Claude et al., 2014) and also decreasing endothelial permeability (Milenkovic et al., 2018). These observations suggest that the observed cognitive and neurological-protective effects of flavanols may be due to their capacity to protect brain-endothelial integrity and BBB permeability.

The objective of this study was to investigate the biological effects of major gut microbiome-derived metabolites of epicatechin, 5-(4′-Hydroxyphenyl)-γ-valerolactone-3′-sulfate and 5-(4′-Hydroxyphenyl)-γ-valerolactone-3′-O-glucuronide, in TNF-α-stimulated human brain microvascular endothelial cells at low concentrations and times of exposure by evaluating their multi-omic modification, including changes in the expression of protein-coding genes, non-coding microRNA (miRNA) and long non-coding RNA (lncRNA) genes together with proteomics modifications.

## Materials and Methods

### Compounds

Epicatechin gut microbiota metabolites: 5-(4′-Hydroxyphenyl)-γ-valerolactone-3′-sulfate (γVL3’S) and 5-(4′-Hydroxyphenyl)-γ-valerolactone-3′-O-glucuronide (γVL3’G) were gifted by Mars, Inc. Chemical structures are presented in Supplementary Figure 1. Stock solutions of γ-valerolactones were prepared by dissolving them in 50% ethanol at 2 mM and stored at −80°C until assayed. For the cell treatments, a mixture of compounds was used in a proportion of 0.65/0.35 for γVL3′S and γVL3′G, respectively. Once dissolved in the culture medium, the final concentration for γVL3′S was 650 nM and γVL3′G was 350 nM, with a total final concentration of 1 μM.

### Cell Culture

Human brain microvascular endothelial cells (HBMEC) were obtained from Angio-Proteomie (Boston, MA, United States). Cells were cultured in the EBM-2 Endothelial Cell Growth Basal Medium supplemented with 2% fetal bovine serum, 0.4% human fibroblast growth factor, 0.1% human epidermal growth factor, 0.1% insulin-like growth factor, 0.1% vascular endothelial growth factor, 0.1% heparin, 0.1% ascorbic acid, 0.1% gentamicin/amphotericin-B, and 0.04% hydrocortisone, all from Lonza (Walkersville, MD, United States). Cell cultures were maintained at 37°C and 5% CO_2_.

HBMECs were used at passage 4. The cells, 50,000 cells/well, were seeded on 24-well plates (Becton Dickinson, United States) that were coated with collagen (Cell Applications, San Diego, CA, United States). At 80% of confluence, cells were exposed to the mixture of gut metabolites for 20 h. Cells treated with medium containing 0.01% ethanol final concentration were used as a control. After the incubation, the medium was discarded, and inflammatory stress was induced by 4 h-incubation with 1 ng/ml of TNF-α (VALs group) (R&D Systems, MN, United States). Cells treated with medium only and incubated with TNF-α were used as a control (TNF-α group). Negative control cells (control group) were treated with medium and no TNF-α incubation.

### RNA Extraction

Total RNA, including short RNAs as miRNAs, were extracted using Monarch^®^ Total RNA Miniprep Kit (New England BioLabs, United States) following the manufacture’s instruction. Briefly, cells were lysed using a lysis buffer and genomic DNA was removed by centrifugation in gDNA removal columns. RNA was then fixed to the RNA purification column, a step that was followed by successive steps of washing and centrifugations. In the end, total RNA was eluted using nuclease-free water. RNA quality and quantity were verified by agarose gel electrophoresis and determination of the absorbance ratio at 260/280 nm using NanoDrop ND-1000 spectrophotometer (Thermo Fisher Scientific, Wilmington, DE, United States). The total RNA samples were stored at −80°C until used.

### Microarray Analysis of mRNA, miRNA, snoRNA, and lncRNA Expression

For transcriptomics analysis, we used Affymetrix Clariom D array for humans, containing over 6 million probes for protein-coding genes but also protein non-coding genes such as miRNAs, lncRNAs, and small nucleolar RNAs (snoRNAs) (Thermo Fisher Scientific, Santa Clara, CA). RNA (100 ng) was used to prepare cRNA and sscDNA using Thermo Fisher Scientific GeneChip^®^ WT PLUS reagent Kit. SscDNA, in an amount of 5.5 μg, was fragmented by uracil-DNA glycosylase (UDG) and apurinic/apyrimidinic endonuclease 1 (APE 1) and labeled by terminal deoxynucleotidyl transferase (TdT) using the DNA Labeling Reagent that is covalently linked to biotin. Fragmented and labeled ssCDNA samples, in triplicate, were used for hybridization, staining, and scanning by using Thermo Fisher Scientific WT array hybridization protocol following the manufacturer’s protocol, at the UC Davis Genome Center shared resource core. Hybridization of fragmented and labeled ssCDNA samples was done using GeneChip^TM^Hybridization oven and the arrays were washed then stained using GeneChip^TM^ Fluidics Station. The arrays were scanned using GeneChip^TM^ Scanner 3000 7G (Thermo Fisher Scientific, Santa Clara, CA). Quality control of the microarrays and data analysis were performed using Thermo Fisher Scientific Transcriptome Analysis Console software version 4.0.2.

### Quantitative Data Analysis

Pair-wise comparisons between biological conditions were applied using specific contrasts. A correction for multiple testing was applied using Benjamini-Hochberg procedure (BH, Benjamini and Hochberg, 1995, PubMed ID 24913697) to control the False Discovery Rate (FDR). Probes with FDR-adjusted *P* < 0.05 were considered to be differentially expressed between conditions. All raw and normalized data are available in GEO database under accession series number: GSE156116.

### Proteomics Analysis

The global proteomics analysis was performed as previously described (Karim et al., 2019). Briefly, the samples, 4 per group, were prepared by homogenization of the cells in lysis buffer and the protein concentration of the supernatant was measured using Bicinchoninic Acid (BCA) protein assay. One hundred microgram of protein sample was denaturized, precipitated and the supernatant was discarded, and the pellet was air-dried. The proteins were then digested and concentrated and labeled using TMT 10-plex peptide labeling (Thermo Fisher Scientific, Canoga Park, CA, United States). All TMT labeled samples were combined in equal amounts. LC separation was performed using Dionex Nano Ultimate 3000 (Thermo Fisher Scientific) with a Thermo Easy-Spray source. Mass spectra have been collected on a Fusion Lumos mass spectrometer (Thermo Fisher Scientific) in a data-dependent MS3 synchronous precursor selection (SPS) method. Then, the MS1 spectra were assimilated in the Orbitrap, 120 K resolution, 50 ms max inject time, 5 × 105 max inject time. MS2 spectra were acquired in the linear ion trap with a 0.7 Da isolation window, CID fragmentation energy of 35%, turbo scan speed, 50 ms max inject time, 1 × 104 automatic gain control (AGC), and maximum parallelizable time turned on MS2 ions were isolated in the ion trap and fragmented with an HCD energy of 65%. MS3 spectra were acquired in the orbitrap with a resolution of 50K and a scan range of 100–500 Da, 105 ms max inject time, and 1 × 105 AGC.

#### Quantitative Data Analysis

The samples then underwent quantitative measurements using isobaric-labeled LC-MS/MS. Raw data were analyzed using Proteome Discoverer 2.2 (Thermo Fisher Scientific) using the default MS3 SPS method. All MS/MS samples were analyzed using Sequest HT to search all human sequences from Uniprot^1^ and 110 common laboratory contaminants^2^ plus an equal number of reverse decoy sequences assuming the digestion enzyme trypsin. Sequest-HT was searched with a fragment ion mass tolerance of 0.20 Da and a parent ion tolerance of 10.0 PPM. Carbamidomethyl of cysteine and TMT10 plex of lysine were specified in Sequest-HT as fixed modifications. Oxidation of methionine and acetyl of the n-terminus were specified in Sequest-HT as variable modifications.

Quantitate Label Based Quantitation (iTRAQ, TMT, SILAC, etc.) was done using Scaffold Q + (version Scaffold_4.9.0, Proteome Software Inc., Portland, OR) for peptide and protein identifications. Peptide identifications with a decoy false FDR cutoff of less than 0.2% were accepted and if they established with at least two unique identified peptides. Proteins with similar peptides that could not be differentiated based on MS/MS analysis were gathered to satisfy the principles of parsimony. Normalization was performed iteratively (across samples and spectra) on intensities and medians obtained by averaging spectra data following log-transformation and weighted by an adaptive intensity weighting algorithm.

### Bioinformatic Analysis and Multi-Omics Integration

#### Genes, Targets, and Proteins Distribution Plots

Data visualization was performed in Manhattan plots to show chromosomal localizations of differentially expressed transcripts and Venn Diagrams to show all possible logical relationships between multiple omic sets. Venn diagrams were used to visualize the relationships between differentially expressed (DE) protein-coding genes (mRNAs), miRNA targets, lncRNA targets, and DE proteins observed in TNF-α vs. control and VALs vs. TNF-α group comparisons. Both plots, Manhattan and Venn diagram, were built using R software packages, the Sushi package^3^ (Phanstiel et al., 2014) and the VennDiagram package^4^ (Chen and Boutros, 2011), respectively. Conversely, associations between genes and groups were searched using unsupervised hierarchical clustering of the samples and the differentially expressed probes using the Pearson correlation as the distance metric and Ward’s method for agglomeration of clusters. The clustering results were illustrated as a heatmap of expression signals. We used the ClustVis^5^ tool to construct the heatmap (Metsalu and Vilo, 2015).

#### Databases-Predicted miRNA Targets

For each miRNA identified as differentially expressed in TNF-α vs. control and VALs vs. TNF-α group comparisons, we performed a target analysis. miRNA targets were predicted using three prediction databases miRTarBase (Hsu et al., 2011), mirRDB (Chen and Wang, 2020), and TargetScan (Agarwal et al., 2015), and only targets identified in at least two of the databases were considered as putative targets for our analysis. For each database, we always chose the most stringent option among those proposed by the database.

#### Databases-Predicted lncRNA Targets

For each lncRNA identified as differentially expressed in TNF-α vs. control and VALs vs. TNF-α group comparisons, we also performed a target analysis. lncRNA target transcripts were predicted by combining the results of two prediction databases from Rtools web server: lncRRISearch^6^ (Fukunaga et al., 2019) and RNARNA^7^. Targets identified in these two databases were considered as putative targets and used in our analysis. For each database, we always chose the most stringent option among those proposed by the database.

#### Protein-Protein Interactions

To perform Protein-Protein Interaction Networks Functional Enrichment Analysis, we used the STRING database (Szklarczyk et al., 2017)^8^.

#### Transcription Factors Analysis

Transcription factors (TFs) potentially involved in the regulation of the expression of identified mRNAs and whose activity can be affected by epicatechin metabolites were searched using enrichR online tool^9^ (Kuleshov et al., 2016). The TFs were searched within TRRUST^10^ (transcriptional regulatory relationships unraveled by sentence-based text-mining) database, a manually curated database of human and mouse transcriptional regulatory networks that contain 8,444 and 6,552 TF-target regulatory relationships of 800 human TFs and 828 mouse TFs, respectively, that have been derived from 11,237 Pubmed articles.

#### Docking Analysis

Molecular docking analysis was employed to explore the potential interaction/binding between identified transcription factors and cell signaling proteins regulating the activity of the transcription factors identified, and the two metabolites, 5-(4′-Hydroxyphenyl)-γ-valerolactone-3′-sulfate (γVL3′S) and 5-(4′-Hydroxyphenyl)-γ-valerolactone-3′-O-glucuronide (γVL3′G). The 3D structures of metabolites were obtained from the PubChem database^11^ and the three-dimensional structure of the proteins was obtained from the Protein Data Bank (PDB) database. Docking calculations were carried out using Blind Docking server^12^ (Sanchez-Linares et al., 2012).

#### Pathway Analysis

##### Pathway Enrichment Analysis

Cellular pathways for differentially expressed genes and proteins from TNF-α vs. control and VALs vs. TNF-α group comparisons were explored using GeneTrail2^13^ (Backes et al., 2007). The lists of significantly regulated mRNAs, miRNA targets, lncRNA targets, and significantly regulated proteins, separately or together, were used to identify the enrichment of biological categories using an over-representation analysis or gene set enrichment analysis (GSEA) with a threshold fixed at *P* < 0.05 using the Benjamini-Yekuteli correction (as recommended by GeneTrail2). KEGG, Biocarta, and Wiki pathways were evaluated in the analysis.

##### Pathways Network

The pathways identified in the step were used to build a network; two pathways were contemplated interconnected if at least one of the genes were common to both. Networks were built and visualized using Cytoscape software (version 3.7.1)^14^ (Su et al., 2014). Data preparation was performed with the use of several R packages included splitstackshape^15^, data.table^16^, dplyr^17^, and string^18^ ^,19^. Pathway networks were built independently for pathways enriched in each omic layer and pathways identified from a global pathway enrichment analysis, considering all omic layers components together. To identify six pathways with the highest degree (number of connections), the Cytoscape Network Analyzer application was used^20^.

##### Networks of Pathways Related to Endothelial Function

Endothelial function-related pathways were selected to construct a network. The endothelial function pathway-specific network was performed in Cytoscape software. As a first step, we obtained a list of genes involved in selected pathways from Kyoto Encyclopedia of Genes and Genomes (KEGG) database (Kanehisa and Goto, 2000). Subsequently, through a series of intersection and non-intersection functions in R, we obtained the mRNAs and proteins involved in the endothelial function pathways, which were also identified to be differentially expressed in our study. Finally, we identified endothelial function pathway components that were targets of miRNAs or lncRNAs.

#### Multilayer Integration and Representation

##### mRNA, miRNA, lncRNA, Proteins, and Target Interaction Networks

Visualization of the interactions between mRNA-TFs, miRNAs-targets, lncRNA-targets, and protein-protein was performed in Cytoscape software. Separate networks for each omic layer and a global network with all integrated interactions were made. Smaller networks were constructed to represent the miRNA targets, lncRNA targets, and DE proteins intersected with mRNAs DE in our study. We identified quantitatively changed mRNAs, miRNAs, and their targets, lncRNAs, and their targets, together with proteins DE in our study and mapped them to the global map provided in the KEGG pathway database, to include a comprehensive pathway topology.

## Results

### TNF-α Modulates Expression of Protein-Coding and Non-coding Genes and Proteins in HBMEC Cells

**FIGURE 1:**
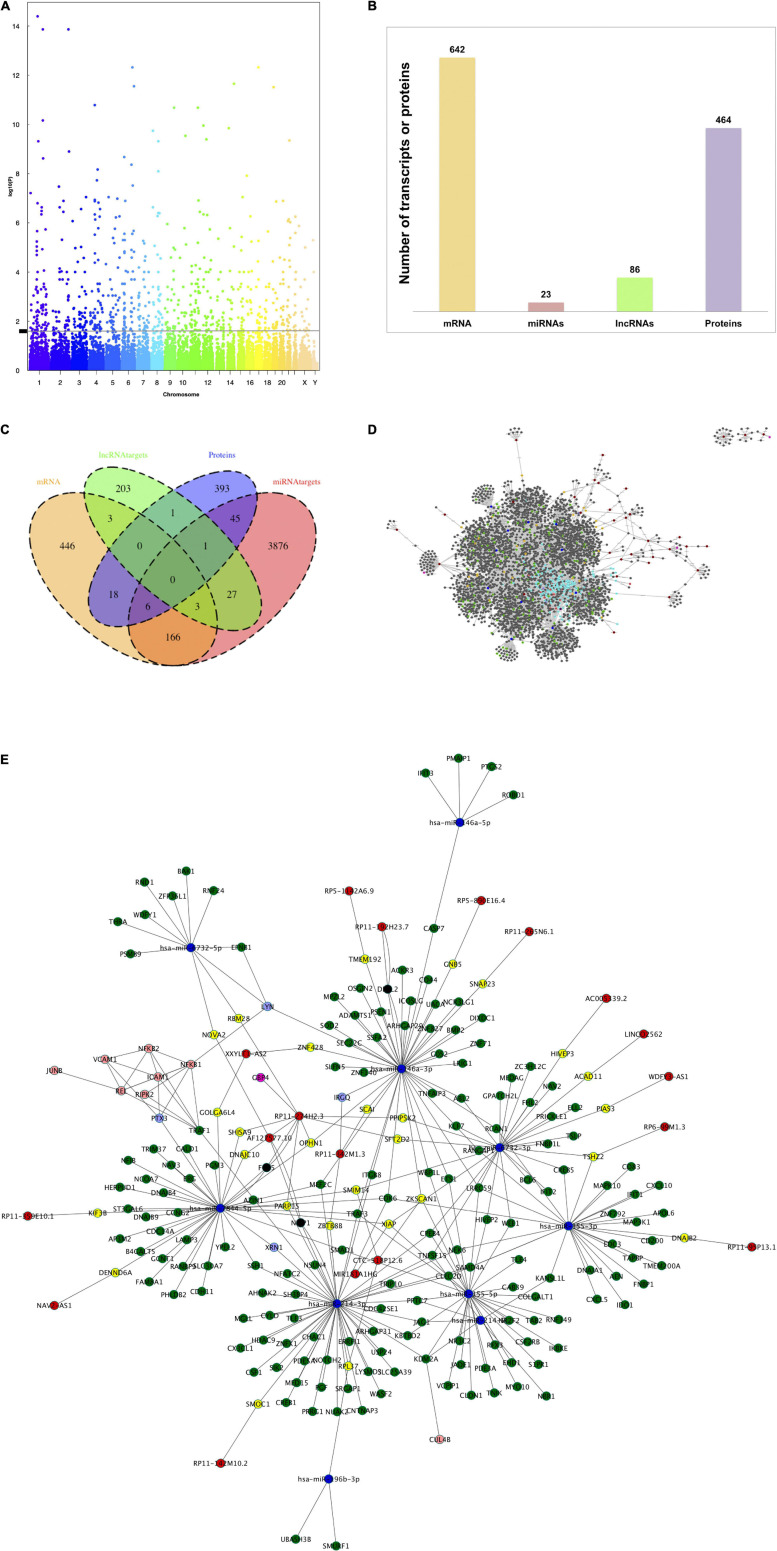
TNF-α modulates the expression of mRNAs, miRNAs, lncRNAs, and proteins in human brain microvascular endothelial cells. HBMEC cells induced under stress by 4 h-incubation with TNF-α were used in microarray analysis and proteomic analysis to determine the differential expression of transcripts and proteins between groups. **(A)** Manhattan plot of total transcripts, 983 transcripts positioned above the line were considered differentially expressed. **(B)** Histogram of the number of differentially expressed mRNAs, miRNAs, lncRNAs, and proteins. **(C)** Venn diagram to indicate the relationships between the number of mRNAs, miRNAs targets, lncRNA targets, and proteins differentially expressed. **(D)** Global network of interactions modulated by TNF-α treatment in HBMEC cells. The total interactions between mRNAs, miRNAs and targets, lncRNAs and targets and protein-protein interactions were used to build a network with Cytoscape software. Nodes colored labels. Red-lncRNAs, blue marine-miRNAs, green-miRNAs targets and mRNAs DE, pink-lncRNA targets and mRNA DE, yellow-lncRNA and miRNA targets and mRNA no DE, purple-mRNAs DE, melon-proteins and mRNA DE, clear purple -proteins and mRNAs DE and regulated by miRNAs or lncRNAs, blue clair-proteins, and mRNAs no DE. DE, differentially expressed. **(E)** Zoom network of interactions between the components of each omic layer and also corresponding to differentially expressed mRNAs. Nodes colored labels. Red-lncRNAs, blue marine-miRNAs, green-miRNA targets, pink-lncRNA targets, yellow-lncRNA, and miRNA targets, melon-proteins, clear purple-proteins regulated by miRNAs, and lncRNAs.

Firstly, we aimed to assess the effect of TNF-α, in comparison to the control group without TNF-α, on the expression of genes, both protein-coding and non-coding transcripts, using transcriptomics microarray, as well as the effect on protein expression using shotgun proteomics. Regarding the effect on gene expression, from 135,751 total probes, 751 were identified as differentially expressed, at least 0.55%, since some probes were discarded during the data cleaning treatment. Manhattan plot showed a uniform distribution of the transcripts on the chromosomes (Figure 1A). We found that of the total differentially expressed transcripts, 85.5% correspond to protein-coding genes (642 mRNAs), 3.1% to miRNAs (23 miRNAs), and 11.4% to lncRNAs (86 lncRNAs) (Figure 1B). Regarding proteomic data, we observed 464 significantly differentially expressed proteins out of a total of 5,565 (Figure 1B).

Subsequently, miRNAs and lncRNAs target gene predictions from the database analysis identified 4125 miRNA targets and 239 lncRNA targets. Thirty-one of these targets were shared between both omic layers (miRNA and lncRNA targets), of which three corresponded to the mRNA and one to the protein category. The number of component intersections between all omic layers is shown in Figure 1C. For instance, 175 miRNA targets belong to the mRNA category, three of them are shared by lncRNA targets and six by proteins, six total lncRNA targets belong to the mRNA category, and 24 total proteins belong to the category of mRNAs.

We extrapolated the nutrigenomic modification data to build a network of interactions, that is considering DE mRNAs, DE miRNA and their targets, DE lncRNA and their targets, and DE proteins. The global network with all the interactions is shown in Figure 1D. The simplified version of the global network is shown in Figure 1E, in which miRNA targets, lncRNA targets, and proteins which belong to DE mRNAs omic layer were considered (at least 196 components as shown in Figure 1C). The components of each category with the highest degree of connections are: (i) miRNAs: hsa-miR-146-3p, hsa-miR-214-3p, hsa-miR-7844-5p, hsa-miR-6732-3p, and hsa-miR-155-5p, (ii) lncRNAs: RP11-274H2.3, RP11-258C19.7, AC098617.1, RP11-373D23.3, RP11-661013.1, (iii) mRNAs: ATP6V1C1, QKI, ANTXR2, TRAF3, TNPO1.

**FIGURE 2:**
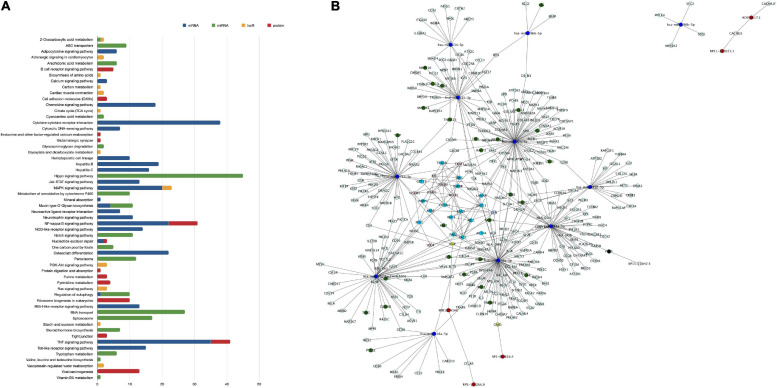
Enriched pathways of differentially expressed transcripts and proteins of human brain microvascular endothelial cells treated with TNF-α vs. control. Differentially expressed transcripts and proteins between study groups were used in a GeneTrial analysis to obtain the list of enriched pathways. Subsequently, a set of genes involved in KEGG endothelial pathway functions was used to map against our differentially expressed transcripts and proteins. **(A)** Histogram that shows the number of transcripts and proteins mapped to the enriched pathways. The blue bars represent the enriched pathways of mRNAs, the green bars of miRNAs, the yellow bars of lncRNAs and the red bars of proteins. **(B)** Network of interactions between mRNAs, miRNAs, and targets, lncRNAs and targets, protein-protein interactions related to a set of genes involved in endothelial cell functions. Node colored labels. Red-lncRNAs, blue marine-miRNAs, green-miRNAs targets and mRNAs DE, yellow-lncRNA and miRNA targets and mRNA no DE, melon-proteins and mRNA DE, clear purple-proteins and mRNAs DE and regulated by miRNAs or lncRNAs, blue clair-proteins and mRNAs no DE, black-miRNA and lncRNA target and mRNA DE, white-miRNA target or lncRNA target and mRNA no DE. DE, differentially expressed.

For functionality analysis, each omic layer’s components were used to perform enrichment analysis and obtain pathways related to mRNAs, miRNA targets, lncRNA targets, and proteins differentially expressed in our study, as presented in the multicolored histogram in Figure 2A. This analysis shows that differentially expressed genes and proteins can impact the cellular functions regulating cell signaling (Toll-like receptor signaling pathways, Ras signaling pathway, NF-kappaB (NF-κB) signaling pathway, or PI3K-Akt signaling pathway), cell-cell adhesions (cell adhesion molecules pathway or adherens junctions), chemotaxis (chemokine signaling pathway, cytokine-cytokine receptor interaction) or cellular metabolism (citrate cycle, amino acid metabolism, or sucrose metabolism). Certain pathways were enriched with miRNA targets such as Notch signaling pathways, RNA transport and spliceosome; or from lncRNA targets such as purine metabolism and tight junction. Some pathways such as TNF-α and NF-κB signaling were enriched by components of mRNA and protein categories; 2-Oxocarboxylic acid metabolism by miRNA and lncRNA targets; and regulation of autophagy by mRNA and miRNA components.

Thenceforward, we constructed a network of interactions between mRNAs, miRNA-targets, lncRNA-targets, and proteins mapped to genes of pathways of endothelial related functions, such as focal adhesion, tight/adherens junctions, or actin cytoskeleton organization (Figure 2B). From 1,392 genes involved in endothelial functional pathways, 113 mRNAs and 24 proteins were differentially expressed in our study. Next, endothelial-related genes were used to search for their regulation by miRNAs or lncRNAs, and 402 miRNA regulations and 15 lncRNA regulations were found. Some of these interactions that may be related to endothelial functions were hsa-miR-7844-5p-TRAF3, REL-VCAM1, and VCAM1-ICAM1, for instance. These data suggest that TNF-α can affect EC functions, such as cell adhesion, junctions, or cell signaling, through a multi-level mode of genomic regulations, simultaneously affecting mRNA, miRNA, lncRNAs, and proteins.

### γ-Valerolactones Can Modulate the Expression of Genes and Proteins in HBMEC

**FIGURE 3:**
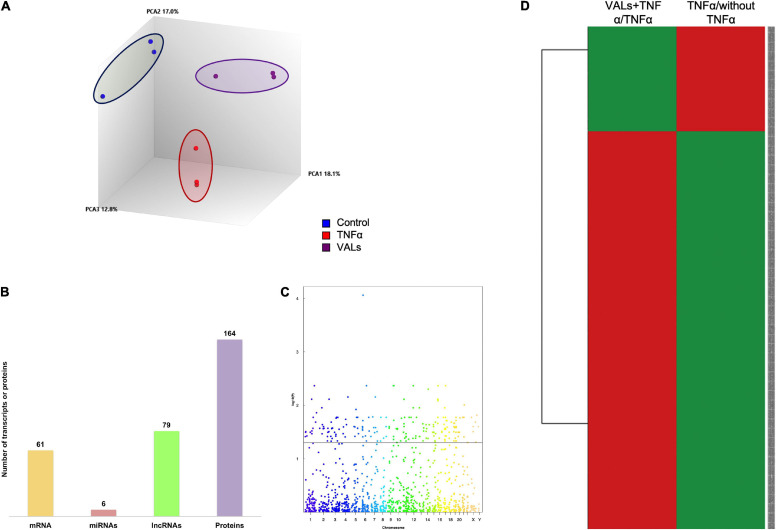
Genomic modifications induced by γ-valerolactones treatment in human brain microvascular endothelial cells. HBMEC cells exposed to the mixture of gut metabolites (γ-valerolactones) for 20 h and induced under stress by 4 h-incubation with TNF-α were used in microarray analysis and proteomic analysis to determine the differential expression of transcripts and proteins between groups, respectively. **(A)** A 3D Principal component analysis (PCA) plot of the data that characterizes the trends exhibited by the expression profiles of HBMEC cells not treated with TNF-α (control, blue), cells treated with TNF-α (TNF-α, red), and cells treated with VALs + TNF-α (VALs, purple). Each dot represents a sample and each color represents the type of the sample. **(B)** Histogram of the number of differentially expressed mRNAs, miRNAs, lncRNAs and proteins. **(C)** Manhattan plot of total transcripts, 211 transcripts positioned above the line were considered differentially expressed. **(D)** Heatmap analyses of gene expression profiles between VALs + TNF-α/TNF-α and TNF-α/without TNF-α. Rows are centered with unit variance scaling applied to rows. Rows are clustered using Euclidean distance and Ward linkage. The levels of gene expression are shown in colors, which transition from green to red with increasing expressions.

To evaluate the effect of γ-valerolactones (VALs) on the transcriptomic and proteomic expression in brain microvascular endothelial cells, a microarray and shotgun analysis were also performed. We compared the global gene expression profiles of the three study groups (Figure 3A) using PLSDA analysis and observed that the VALs groups had different expression profiles from the TNF-α and control groups. This observation suggests changes in expression profile in HBMEC following exposure to the VALs.

Statistical analysis was then performed to identify differentially expressed transcripts. The number of DE transcripts in cells treated with TNF-α + VALs (VALs group) vs. TNF-α alone (TNF-α group) was 164. Integrally, 37.2% of these modifications correspond to protein-coding genes (61 mRNAs), 3.6% to miRNAs (6 miRNAs), 48.2% to lncRNAs (79 lncRNAs) (Figure 3B). On the other hand, proteomics analysis indicates 164 differentiated proteins (Figure 3B). A Manhattan plot of transcripts shows that differentially expressed genes are localized throughout the genomes (Figure 3C). The expression profile of genes identified in the VALs group was compared with the expression profile of genes identified as differentially expressed by TNF-α group and presented by a heat map, Figure 3D. This analysis revealed that the expression of genes obtained following exposure to VALs presents the opposite expression profile when compared to the TNF-α group, that is, genes identified as up-regulated by exposure of cells to TNF-α were identified as down-regulated by VALs and inversely, genes identified as down-regulated by TNF-α were identified as up-regulated by VALs. This observation suggests that exposure of HBMEC to VALs can, at least partially, counteract the inflammatory stress induced by TNF-α.

Furthermore, these data suggest for the first time that γ-valerolactones, one of the epicatechin gut microbiota metabolites, can exert complex genomic modification. These effects, however, seem to be of a lesser impact on genomic and proteomic modifications than of the TNF-α effect.

#### γ-Valerolactones Modulate the Expression of Protein-Coding Genes in HBMEC

**FIGURE 4:**
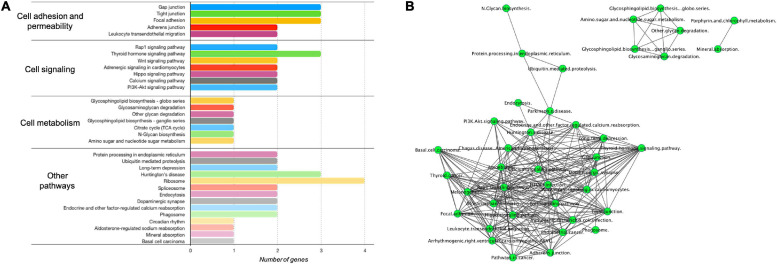
Enriched pathways of protein-coding genes differentially expressed in human brain microvascular endothelial cells treated with γ-valerolactones. Differentially expressed protein-coding genes between VALs and TNF-α groups were used in a GeneTrial analysis to obtain the list of enriched pathways. **(A)** Histogram that shows the number of genes mapped to the enriched pathways classified in cell adhesion and permeability, cell signaling, cell metabolism and other pathways. **(B)** Pathway interactions network built in Cytoscape software to show the connections (edges) between enriched pathways (nodes) by differentially expressed protein-coding genes.

To seek the functional pathways that could be modulated by DE protein-coding genes (Supplementary Table 1) in our VALs study group vs. the TNF-α group, we performed an enrichment analysis in GeneTrial. This analysis showed that mRNAs are involved in different processes regulating cell adhesion and permeability (Gap junction, Tight junction, Focal adhesion, Adherens junction, Leukocyte transendothelial migration pathways), cell signaling (Rap1 signaling, Thyroid hormone signaling, Wnt signaling, PI3K-Akt signaling), cell metabolism (Glycosphingolipid biosynthesis, TCA cycle, Amino sugar, and nucleotide sugar) and other pathways, as shown in Figure 4A. Subsequently, we constructed a network to integrate and show connections between the pathways resulting from our previous analysis (Figure 4B). Three clusters were observed, the largest including Rap1, Wnt, and PI3K-Akt signaling pathways strongly connected with leukocyte transendothelial migration, adherens junction, tight junction, and gap junction. The two smallest consist of a cluster that involves glycosphingolipid biosynthesis, amino sugar and nucleotide sugar metabolism and glycosaminoglycan degradation. We can observe a separation of the processes related to cellular metabolism and those of cell-cell contact regulation. The pathways with the highest degree of interaction with other pathways are Wnt signaling, Rap1 signaling, tight junction, and hippo signaling pathway. Consequently, this observation suggests that γ-valerolactones exposure could modulate endothelial cell function, that is adhesion with immune cells but also endothelial cell permeability, by regulation of mRNAs, miRNAs, lncRNAs, and proteins involved in these processes.

#### γ-Valerolactones Modulate the Expression of miRNA in HBMEC

**FIGURE 5:**
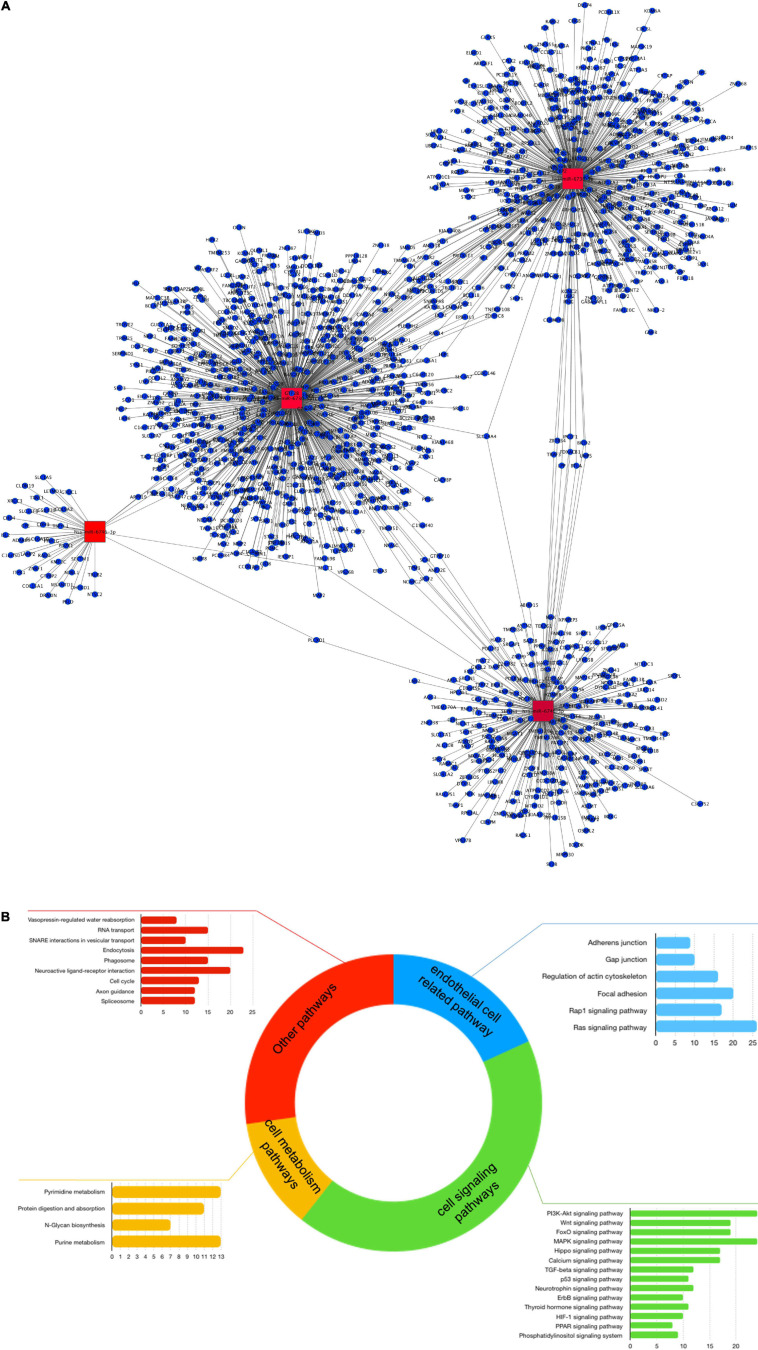
MiRNAs expression mediated by γ-valerolactones treatment in human brain microvascular endothelial cells and their relationship with endothelial cell functions. HBMEC cells exposed to the mixture of gut metabolites (γ-valerolactones) for 20 h and induced under stress by 4 h-incubation with TNF-α were used in microarray analysis to determine the differential expression of miRNAs between groups. Subsequently, the miRNAs targets were obtained from an analysis of databases (miRTarbase, TargetScan, and miRDb). An enrichment pathway analysis of miRNAs targets in GeneTrial was also performed. **(A)** Network of miRNAs differentially expressed and their targets. A network built-in Cytoscape software. Hubs network components are highlighted by red squares. **(B)** Histogram that shows the number of genes mapped to the enriched pathways classified as endothelial cell-related (blue), cell signaling (green), cell metabolism (yellow), and other pathways (red).

MiRNA expression was also identified as modulated following exposure of HBMEC to γ-valerolactones, suggesting the capacity of these metabolites at low concentrations to modulate the expression of small non-coding RNAs (Supplementary Table 2). The search for their putative targets from the databases for six differentially expressed miRNAs, hsa-miR-6730-3p, hsa-miR-6730-5p, hsa-miR-3661-3p, hsa-miR-3661-5p, hsa-miR-6746-3p, hsa -miR-6746-5p, allowed us to identify 1360 target genes, including ARSB, CXADR, FREM1, or HAP1. Among them, five targets are in common with differentially expressed mRNAs (Supplementary Figure 3A). Figure 5A shows the network topology of miRNA-target interactions which revealed at least four miRNA hubs for the hsa-miR-6730-5p, hsa-miR-6730-3p, hsa-miR-6746-5p, and has-miR-6746-3p.

Likewise, miRNA target functionality analysis was performed by placing these genes into cellular pathways. MiRNA targets have been found to play a role in pathways such as those regulating EC functions (adherens junction, gap junction, focal adhesion), cell signaling (PI3K-Akt, Wnt, Foxo, MAPK, PPAR signaling), or cell metabolism (pyrimidine, purine, and protein digestion metabolism) (Figure 5B). 36 resulting pathways were found in turn in mRNAs enrichment analysis (Supplementary Figure 3B), this suggests that although only a few targets are shared in both omic layers (mRNA and miRNA targets), their functionality is similar. In Supplementary Figure 2, the connections between miRNA targets-related pathways are shown, where centrality is one of its characteristics. We observed that the pathways with the highest degree of interaction with others are pathways in the Ras signaling pathway, PI3K-Akt signaling, FOXO signaling, viral carcinogenesis, and MAPK signaling pathway.

#### lncRNA Expression Modulation by γ-Valerolactones

**FIGURE 6:**
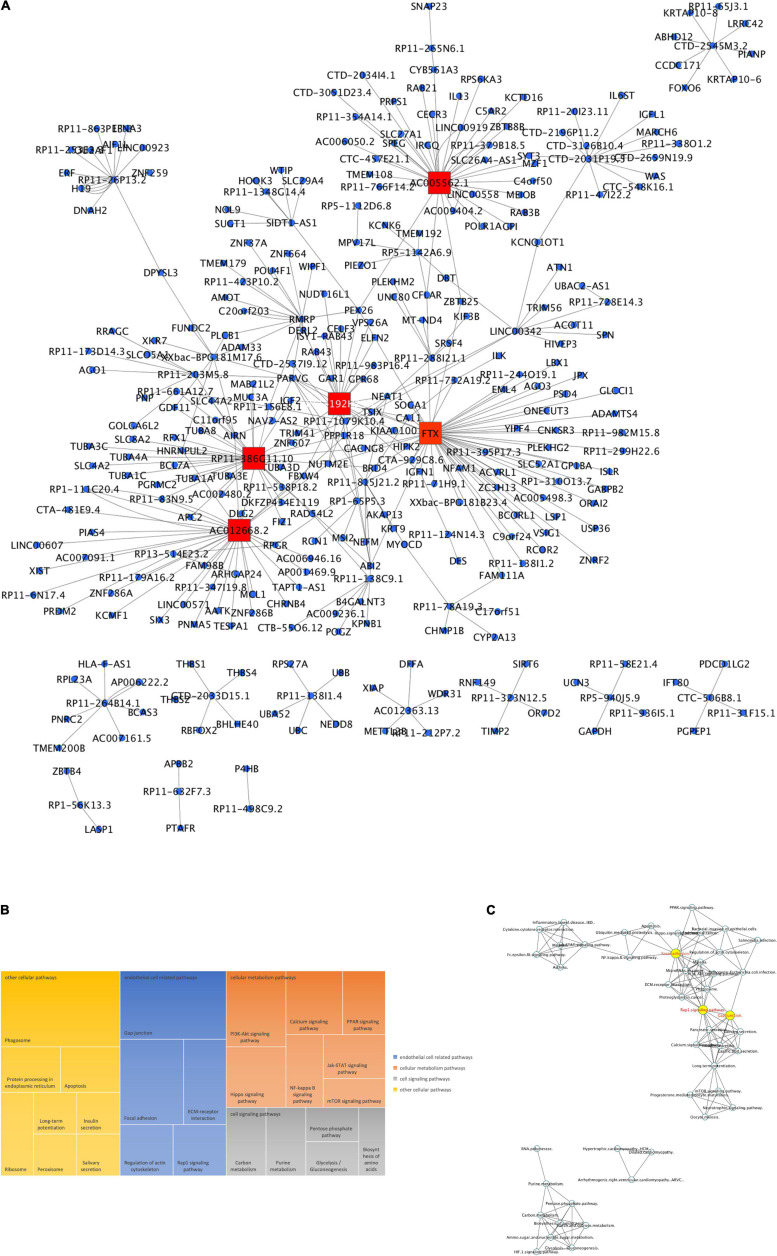
LncRNAs expression mediated by γ-valerolactones treatment in human brain microvascular endothelial cells and their relationship with endothelial cell functions. HBMEC cells exposed to the mixture of gut metabolites (γ-valerolactones) for 20 h and cells induced under stress by 4 h-incubation with TNF-α were used in microarray analysis to determine the differential expression of lncRNAs between groups. Subsequently, the lncRNAs targets were obtained from an analysis of databases (RNARNA, LncRRIsearch). An enrichment pathway analysis of miRNAs targets in GeneTrial was also performed. **(A)** Network of lncRNAs differentially expressed and their targets. A network built-in Cytoscape software. Hubs network components are highlighted by red squares. **(B)** lncRNA targets were overrepresented by genes coding for KEGG, Biocarta and Wiki modules associated with endothelial cell-related, cellular metabolism, cell signaling and other pathways. **(C)** lncRNA-enriched pathway interactions network. Pathway interactions network built in Cytoscape software to show the connections (edges) between enriched pathways (nodes) by lncRNAs differentially expressed. Hubs network components are highlighted by yellow circles.

Microarray analysis has also shown for the first time that exposure of HBMEC to γ-valerolactones can also modulate the expression of another group of protein non-coding RNA, which are long-non-coding RNAs. We observed a change in expression of 79 lncRNAs (Supplementary Table 3). A search of databases for their targets allowed us to identify 364 lncRNA-targets. Among these 364 targets, 4 (UBC, C11orf95, PLCB1, BHLHE40) are in common with protein-coding genes and 14 (IL6ST, ABHD12, HIPK2, PLEKHG2, PSD4, ZNF37A, AMOT, POU4F1, UBC, CELF3, AGO1, EFNA3, BCL7A, PRDM2) with miRNAs targets (Supplementary Figure 3A). In Figure 6A lncRNA-targets interactions topology is shown which identified several clusters of genes, such as for lncRNA RP11-386G11.10, RP11-192H23.7, AC012668.2, FTX, AC005562.1, or lncRNAs hubs.

In lncRNAs targets functionality analysis, we observed an enrichment of pathways involved in the regulation of EC (gap junction, focal adhesion, ECM-receptor interaction, regulation of actin cytoskeleton, Rap1 signaling pathways), cellular metabolism (PI3K-AKT, calcium, PPAR, NF-κB, JAK-STAT, mTOR signaling pathways), or cell signaling (carbon, purine, pentose phosphate, glycolysis, gluconeogenesis metabolism) (Figure 6B). We can corroborate that most of the pathways described are themselves the result of mRNA and miRNA targets pathway analysis. Sixteen pathways were shared with protein-coding genes enrichment analysis, while 37 are shared with miRNAs pathways analysis (Supplementary Figure 3B).

To analyze the connections between these pathways, we built a network of interactions presented in Figure 6C. We observed a strong relationship between intracellular signaling pathways, PPAR, NF-κB, PI3K-Akt, Rap1 signaling, with focal adhesion and gap junction. These pathways were grouped in a large cluster, connected with a smaller cluster in which inflammatory and cytokine pathways were involved. Metabolic processes were grouped in a separate cluster. The pathways with the highest degree of connections with other pathways are Rap1 signaling, Focal adhesion, long-term potentiation, microRNAs in cancer, phagosome, and gap junction. Taken together, this study shows for the first time the capacity of γ-valerolactones to modulate the expression of miRNAs and lncRNAs, particularly those involved in the regulation of endothelial cell function and permeability.

#### Proteomics Modulation by γ-Valerolactones

**FIGURE 7:**
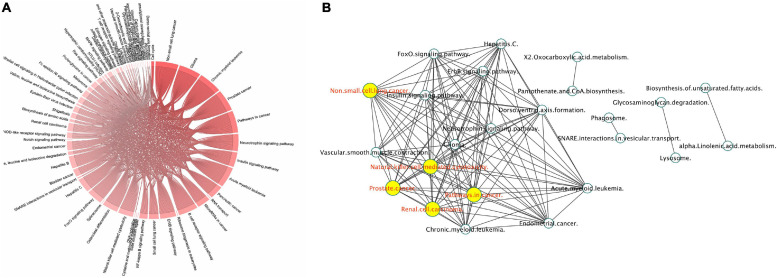
Protein expression mediated by γ-valerolactones treatment in human brain microvascular endothelial cells and their relationship with endothelial cell functions. HBMEC cells exposed to the mixture of gut metabolites (γ-valerolactones) for 20 h and cells induced under stress by 4 h-incubation with TNF-α were used in a shotgun proteomic analysis to determine the differential expression of proteins between groups. An enrichment pathway analysis of miRNAs targets in GeneTrial was also performed. **(A)** Proteins differentially expressed mapped onto KEGG, Biocarta, and Wikipathways modules associated with endothelial cell function, cell metabolism, cell signaling pathways, and others. **(B)** Proteins-enriched pathway interactions network, pathway interactions network built in Cytoscape software to show the connections (edges) between enriched pathways (nodes) by proteins differentially expressed. Hubs network components are highlighted by yellow circles.

The use of the proteomic untargeted shotgun approach allowed us to demonstrate that exposure of HBMEC to γ-valerolactones can also affect the expression of proteins (Supplementary Table 4). We observed that γ-valerolactone metabolites modulated the expression of 164 different proteins. Functional analyses revealed that these proteins play a role in pathways related to cell metabolic pathways, cell signaling pathways, and others, as shown in Figure 7A. In metabolic processes, we observed that valine, leucine, and isoleucine biosynthesis, 2-oxocarboxylic acid, biosynthesis of unsaturated fatty acids, and glycosaminoglycan degradation were part of this category. The interactions between pathways are shown in Figure 7B. The pathways with the highest degree of connections with other pathways were renal cell carcinoma, prostate cancer, or natural killer cell-mediated cytotoxicity.

#### Transcription Factor and Interactome Analysis Using 3D *in silico* Modeling for γ-Valerolactones

**FIGURE 8:**
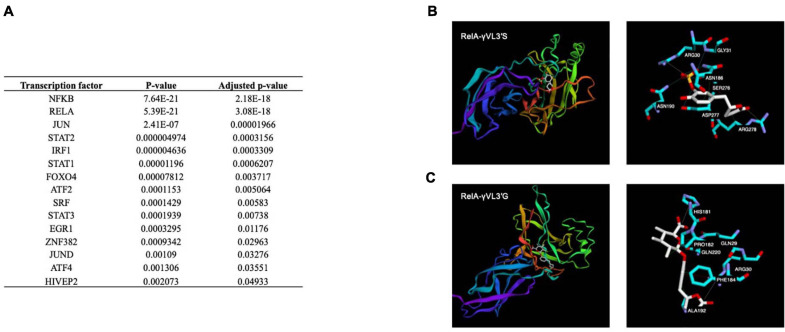
Transcription factors potentially involved in the nutrigenomic modifications induces by γ-valerolactones and their *in silico* interaction with RelA subunit of NF-κB. **(A)** List of potential transcription factors identified using bioinformatic analysis. Binding mode obtained by computational docking for: **(B)** 5-(4′-Hydroxyphenyl)-γ-valerolactone-3′-sulfate, and **(C)** 5-(4′-Hydroxyphenyl)-γ-valerolactone-3′-O-glucuronide.

Using gene expression analysis data, the Ttrust database was searched using the EnrichR tool to identify potential TFs whose activity could be affected by γ-valerolactones and involved in the observed changes in the expression of genes. Among the most significant TFs identified were NF-κB1, cJUN, STAT2, IRF1, or FOXO4 (Figure 8A). Following this analysis, we aimed to identify if γ-valerolactones could have a binding affinity with these TFs, binding that may affect their activity and consequently result in changes in expression of genes as we observed, under the hypothesis that these metabolites can enter into the cells. Using this approach, we observed that γ-valerolactones have the potential to bind to a transcription factor, an interaction that could affect their activity and induce changes in the expression of genes, as observed using our microarray analysis. The analysis suggests that the glucuronidated form of γ-valerolactone has a slightly higher potential to bind to protein when tested than sulfated metabolite. The highest binding was observed between γVL3′G and RelA protein, with binding free energy identified being −7 kcal/mol (Figure 8B), followed by its binding to NF-κB (−6.9 kcal/mol). γVL3′S showed the highest binding capacity with RelA (binding energy of −6.6 kcal/mol) (Figure 8C).

### Integration of Multi-Omics Data of γ-Valerolactones Treatment in HBMEC Cells

**FIGURE 9:**
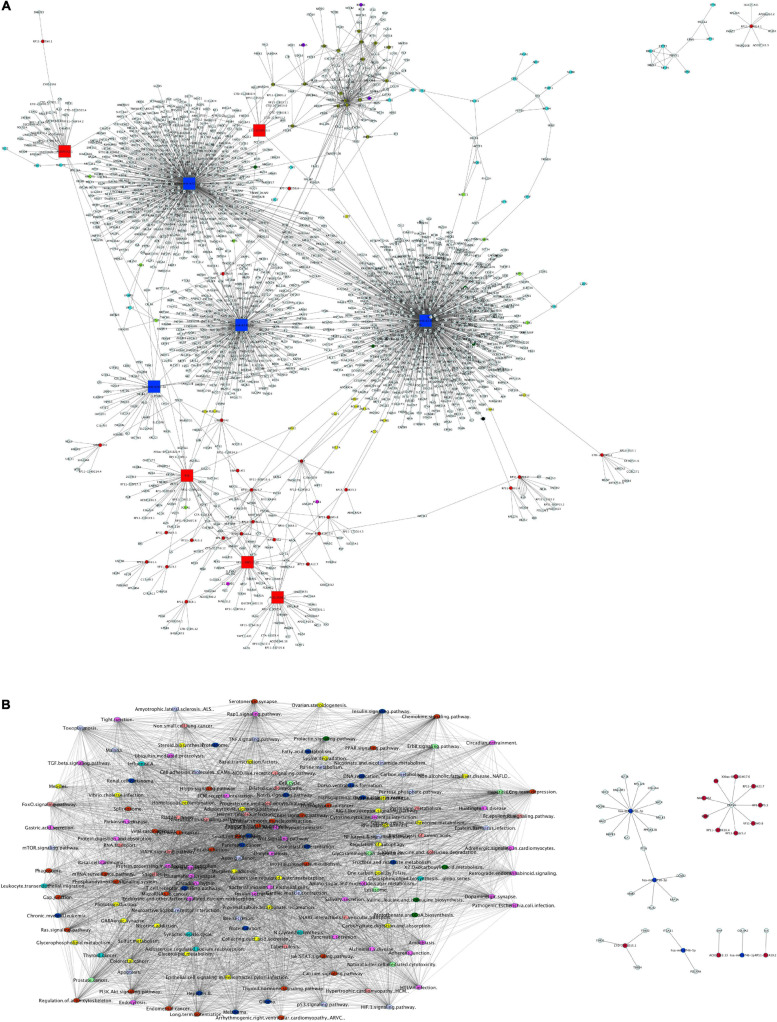
Global network of interactions modulated by γ-valerolactones treatment in human brain microvascular endothelial cells and their relation with endothelial function pathways. **(A)** The total interactions between mRNAs, miRNAs, and targets, lncRNAs and targets and protein-protein interactions were used to build a network with Cytoscape software. Nodes colored labels. Red-lncRNAs, blue marine-miRNAs, green-miRNAs targets and mRNAs DE, pink-lncRNA targets and mRNA DE, yellow-lncRNA and miRNA targets and mRNA no DE, purple-mRNAs DE, blue clair-proteins and mRNAs no DE, black-miRNA and lncRNA target and mRNA DE, clair green-proteins and mRNA no DE and regulated by miRNA or lncRNA, olive-TFs, white- components not belonging to any of the previous color categories. DE, differentially expressed. Hubs network components are highlighted by bigger squares. **(B)** mRNA, miRNA targets, lncRNA targets and proteins were used to perform global GeneTrial enrichment analysis and obtained the pathways related. A pathway-connections network was built in Cytoscape. Node colored labels represent the omic layers (mRNA, miRNA targets, mRNA targets, proteins or combinations) from which the pathways were enriched. Yellow-miRNA, green-proteins, pink-mRNA + lncRNA + miRNA, blue marine-miRNA + proteins, clair blue-mRNA + miRNAs, clair purple-lncRNA + miRNA, red-mRNA + miRNA + lncRNA + proteins, melon-lncRNA + miRNA + proteins, clair green-mRNA + miRNA + proteins. **(C)** Small network of focal adhesion components and regulated by miRNAs and lncRNAS differentially expressed in our VALs vs. TNF-α groups comparison.

As a first step toward data integration, we grouped mRNA, miRNA targets, lncRNA targets, and protein interactions from their analyses into a global network of VALs vs. TNF-α comparison. The network presented in Figure 9A shows at least three main clusters, dominated by hsa-miR-6730-3p, hsa-miR-6730-5p, hsa-miR-6746-3p, and hsa-miR-6746-5p. Likewise, lncRNA smaller clusters are observed, formed by FTX, RP11-386G11.10, AC012668.2, AC005562.1, CTD-2031P19.5 as central hubs. Moreover, the global network of enriched pathway interactions of our omic layers (mRNAs, miRNAs, lncRNAs, and proteins) was grouped and shown in Figures 9B,C, where individual clusters were not differentiated, but rather a centralized network. The pathways with the highest degree of connections with other pathways into the network are pathways in cancer, focal adhesion, thyroid hormone signaling pathway, RAS signaling, PI3K-Akt signaling, and Rap1 signaling.

**FIGURE 10:**
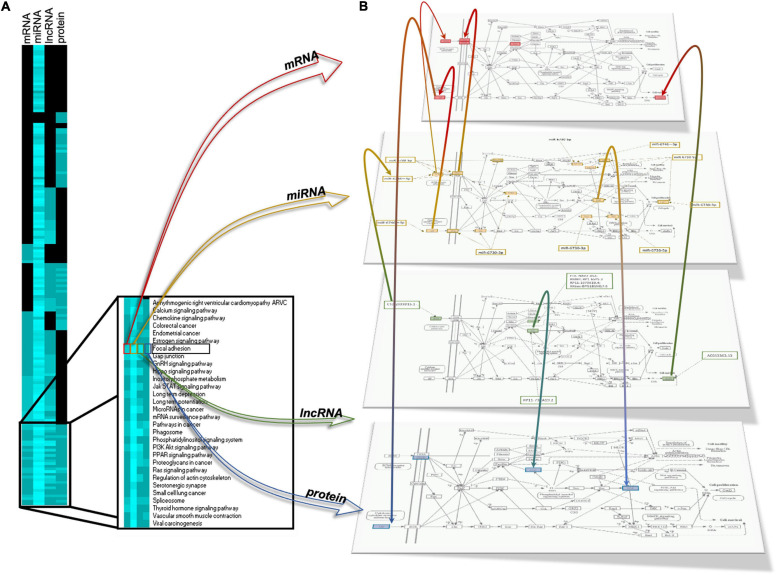
Integration of differentially expressed mRNAs, microRNA target gene, lncRNA target gene and protein modulating focal adhesion function in human brain microvascular endothelial cells treated with γ-valerolactones and stressed with TNF-α. **(A)** Heatmap of differentially expressed gene pathways, miRNA target gene pathways, lncRNA gene pathways and protein pathways. The intensity of the blue color depends on the number of genes in each pathway, the lighter blue color is indicative of a higher number of genes. Comparisons of pathways between biological categories identified a group of pathways in common including chemokine signaling pathway, cytokine cytokine receptor, focal adhesion, gap junction, FOXO signaling, fatty acid metabolism, and pathways that regulate endothelial cell interaction and permeability. **(B)** A representative integrated analysis of differentially expressed genes and proteins, and target genes of differentially expressed miRNAs, lncRNA is shown for Focal adhesion. Yellow = differentially expressed genes; Red = target genes of differentially expressed miRNAs, Green = target genes of differentially expressed lncRNAs, Blue = differentially expressed proteins. Color gradations = genes identified in both omic maps.

Our next step was to identify common enriched pathways to mRNAs, miRNAs, lncRNAs, and proteins. As shown in the heatmap in Figure 10A, pathways specific to one, two, or three omic analyses (mRNA, miRNA, lncRNA) were identified but also a group of pathways, 30, have been identified as common for all the four omics analyses (mRNA, miRNA, lncRNA, and proteins). Among these pathways are gap junction, regulation of actin cytoskeleton chemokine signaling pathway, PPAR signaling, PI3K signaling, Ras signaling or focal adhesion (Figure 10A, side box). Figure 10B provides an example of integration for the focal adhesion pathway showing the integrated analysis of the four regulatory layers, that is mRNA, miRNA, lncRNA, and protein regulation, with their interactions, revealing how this pathway can be modulated by γ-valerolactones. Some genes involved in focal adhesion are regulated by miRNAs and lncRNAs, such as Integrin beta (ITGB), found in protein, mRNA, and miRNA map and regulated by hsa-miR-6746-3p. Integrin-linked protein kinase (ILK) found in the protein and lncRNA map is being regulated by RP11-732A19.2. Phosphatase and tensin homolog (PTEN) was found in the miRNA map and regulated by miR-6730-5p and miR-6730-3p. Protein kinase (PAK) protein is regulated by miR-6730-5p. Taken together, this integrated multi-omic analysis suggests that genomic modifications induced by γ-valerolactones highly impact pathways regulating endothelial cell function and permeability.

## Discussion

The BBB has an important role in the health of the brain and is often compromised in disease. The BBB is composed of BMECs and other cells such as astrocytes and matrix molecules that help regulate the movement of immune cells and molecules into and out of the brain. Complex intercellular tight junctions limit the passive diffusion of molecules into the brain (Lippmann et al., 2012). Neuroinflammation together with mitochondrial dysfunction are found in chronic neurodegenerative diseases. Both can lead to higher oxidative stress which can promote neuronal damage and consequently inflammation that results in a feed-forward loop of neurodegeneration. Natural compounds, especially polyphenols, have proven capable of modifying different neuropathological features, such as in AD. Attention has therefore been given to flavan-3-ols, flavonoids abundant in cocoa, tea, red wine, berries, and other plant-derived foods and beverages. Phenyl-valerolactones (PVL) are the major group of circulating flavan-3-ol metabolites in humans (Ottaviani et al., 2016), metabolites that can cross the blood-brain barrier (Angelino et al., 2019).

**FIGURE 11:**
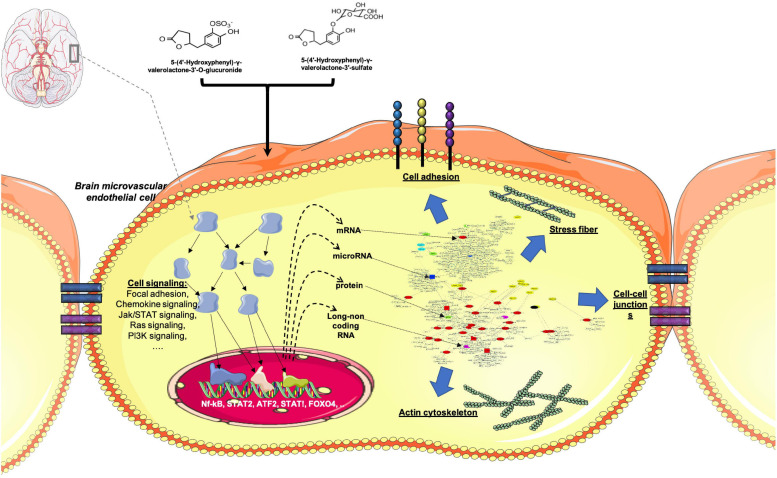
Conceptual summary of genomic modification of γ-valerolactones in human brain microvascular endothelial cells. Differential expression of protein-coding genes, microRNAs, lncRNAs, and proteins that could regulate cellular processes such as those regulating brain microvascular endothelial cells permeability. Adapted from BioRender.com.

In this study we assessed the effect of γ-valerolactones treatment on HBMEC cells on the inhibition of TNF-α -generated stress *in vitro*, through the multi-omic data analysis that included mRNAs, miRNAs, lncRNAs, and protein layers integration (Figure 11). We observed that TNF-α alone exerted greater genomic modifications on transcript and protein modulation than treatment with TNF-α + γ-valerolactones (VALs), which could indicate that the pro- inflammatory effect of TNF-α could be corrected by these metabolites. We performed an analysis of differential expression and pathways associated with each omic data category individually and globally. After the treatment with VALs, we assessed that miRNA targets-mapped pathways are more associated to cell signaling and those protein-mapped pathways are more related with metabolism. However, when we performed the omic integration analysis, we observed a strong association between cell adhesion and permeability pathways, such as focal adhesion, tight junction, gap junction, adherens junctions with cell signaling pathways, such as Rap1, PI3K-Akt, Wnt, and thyroid hormone signaling. Signaling pathways such as Rap1, Wnt, and PI3K-Akt, have been found to play a crucial role in the maintenance of cell-cell regulation (Jasaitis et al., 2012; Shah et al., 2018).

Our analysis allowed us to extract some key elements and their target interactions in the modulation of EC function pathways. Here, we identified mRNAs, miRNAs, lncRNAs, and proteins involved in cell adhesion and permeability functions. miRNAs are short ncRNAs with a length of 19–23 nucleotides. MiRNAs have two main functions: post-transcriptional gene regulation and RNA silencing. Consequently, the mRNAs can be regulated by one or more mechanisms including inhibition of mRNA translation to proteins by ribosomes and by miRNA cleavage that results in mRNA disruption (Hernandez-Romero et al., 2019). Clues about brain endothelial function modulated by miRNAs such as hsa-miR-146a-3p, hsa-miR-214-3p in the TNF-α group and hsa-miR-6746 and miR-6730-5p in the VALs group were obtained in our analysis. Deng et al. (2017), showed that miR-146a was upregulated in lineage negative bone marrow cells in aged mice, which were enriched in endothelial progenitor cells (EPCs) (Deng et al., 2017). An increase in expression of miR-146a increases senescence and apoptosis, suggesting that miR-146a inhibition can improve vascular repair in EPCs (Deng et al., 2017). On the other hand, stimulation of primary Human Umbilical Vein Endothelial Cells (HUVECs) with lipopolysaccharide (LPS) induced expression of miR-146a in replicative senescent HUVECs (Olivieri et al., 2013). Therefore, the increase of miR-146a in ECs can represent senescence-associated pro-inflammatory conditions in the vasculature. *In vitro*, TNF-α, and IFNγ treatment of human cerebral microvascular endothelial cells resulted in upregulation of miR-146a, which is in agreement with our data (Wu et al., 2015). On the other hand, miR-214, another miRNA found in the TNF-α group, is suggested as a biomarker to detect early stages of Parkinson’s disease (Dong et al., 2016).

Hsa-miR-6746 is a miRNA found upregulated in the VALs group, this miRNA has been studied in different contexts, but scarce information exists about its relationship with EC function. It would be interesting to extrapolate the epigenetic mechanisms attributed to this miRNA to study the function of brain ECs. In this regard, it has been reported that the splicing activator protein SRSF2 and the splicing inhibitor protein HNRNPD may be implicated in EC senescence. EC senescence has also been associated with vascular dysfunction and increased vascular risk. Our observations demonstrated that SRSF2 can be targeted by miR-6730-5p and miR-6746-5p, two miRNAs differentially expressed in the VALs group, which could indicate a mechanism through which VALs prevent EC dysfunction.

LncRNAs are protein non-coding RNAs longer than 200 nucleotides and are categorized depending on proximity to protein-coding genes, such as intergenic, intronic, bidirectional, sense, and antisense lncRNAs (Fang and Fullwood, 2016). The function of a lncRNA is to act as a molecular signal and regulate transcription in response to various stimuli (Fang and Fullwood, 2016). LncRNAs have tissue-specific expression profiles and are mainly situated in the nucleus and less in the cytoplasm. There is evidence suggesting that lncRNAs are more enriched within chromatin than miRNAs (Hernandez-Romero et al., 2019). In our analysis, we identified lncRNAs differentially expressed, such as FTX, RP11-386G11.10, RP11-192H23.7, AC012668.2, AC005562.1 found in the VALs group, and searched their connections in brain EC function. For instance, five primes to Xist (FTX) is downregulated in VALs group, and it was shown that overexpression of FTX inhibited apoptosis in H_2_O_2_ treated cardiomyocyte and ischemia-reperfusion (I/R) injury mice model by negatively regulating miR-29b-1-5p (Li et al., 2019). miR-29 is needed for normal endothelial function and has therapeutic cardiometabolic potential (Widlansky et al., 2018). Therefore, downregulation of FTX could contribute to the upregulation of miR-29 and subsequently to an improvement in EC function.

Expression of peroxisome proliferator-activated receptor γ (PPARγ) was positively correlated with lncRNA FTX (Li et al., 2018). PPARγ overexpression in brain endothelial cells induces decreased inflammation-induced ICAM-1 and VCAM-1 upregulation and subsequent adhesion and transmigration of T cells. Therefore, it has been proposed that PPARγ in brain endothelial cells can be exploited to target harmful EC-T cell interactions in inflammatory conditions (Klotz et al., 2007). Moreover, HIV-1 neuropathogenesis increased adhesion, and migration of HIV-1 infected monocytes across BBB were significantly lowered when bovine brain microvascular endothelial cells (BMVEC) were treated with PPARγ agonist. These findings suggest that PPARγ agonists can be a novel approach for the treatment of neuroinflammation by preventing monocyte migration across the BBB (Ramirez et al., 2008). FTX, although down-regulated in our study, appears to be related to PPARγ, and PPARγ signaling was one of the enriched pathways in our global enrichment analysis. Therefore, diverse mechanisms must converge for PPARγ to be activated in the VALs group.

Likewise, we identified TFs regulating our mRNA omic layer. NF-κB was one of the transcriptional factors found in our analysis and was found to regulate some of the differentially expressed transcripts. NF-κB activation initiates the canonical and non-conical pathways that promote activation of TFs leading to inflammation, such as leukocyte adhesion molecules, cytokines, and chemokines. However, flavonoids may control the expression of pro-inflammatory genes leading to the attenuation of the inflammatory responses of various pathologies (Choy et al., 2019). It has been corroborated that small molecules derived from dietary (poly)phenols may cross the BBB, get to brain cells, moderate microglia-mediated inflammation, and exercise neuroprotective effects, with possible alleviation of neurodegenerative diseases. The polyphenol metabolites can lower neuro-inflammatory processes via regulation of nuclear factor NF-κB translocation into the nucleus and modulation of IκBα (inhibitor of NF-κB) levels (Figueira et al., 2017). Our *in silico* docking analysis also suggests that valerolactones metabolites present the capacity to bind to this transcription factor, binding that could affect its activity and consequently the expression of related genes. This analysis also suggested that the studied valerolactone metabolites present a potential binding capacity to different transcription factors. Earlier studies have suggested that epicatechin secondary metabolites can bind to these cell signaling proteins (Milenkovic et al., 2018) but it is the first to suggest such interaction for gut metabolites.

By omic layers integration, our analysis allowed us to find relationships between HBMEC and focal adhesion pathways. We were able to identify components mapped to the focal adhesion network, which were differentially regulated in our study (Figure 10). For instance, alpha integrin (ITGA) and ITGB and their relationship with Platelet endothelial cell adhesion molecule (PECAM1-1). PECAM-1 is involved in controlling BBB permeability and although not necessary for T-cell diapedesis, it is of importance for the cellular route of T-cell diapedesis across the BBB (Wimmer et al., 2019). In EC, this molecule regulates junctional and adhesive properties. It is reported that in an inflammatory condition, such as in vessels affected by atherosclerosis, the function of PECAM-1 is compromised which can lead to augmented adhesion of immune cells to EC, diminished vascular integrity, and greater leukocyte transmigration to the intima-media. PECAM-1 contains six extracellular Ig-like domains that mediate the attraction and adhesion of leukocytes to EC, such as augmenting eosinophil adhesion to IL-4-stimulated HUVECs in an α4β1 integrin-dependent manner (Chistiakov et al., 2017). Therefore, searching for mechanisms that mediate PECAM-1 interactions with beta or alpha integrins is of importance. Hsa-miR-6746-3p and hsa-miR-6730-5p were two differentially regulated miRNAs in the VALs group whose targets include ITGA and ITGB. In conditions of stress, such as TGF-β stimulus, the inhibition of these two integrins could prevent leukocyte transendothelial migration (TEM) under inflammatory conditions.

Another possible mechanism of VALs function against TNF-α inflammation could be mediated by Integrin-linked kinase (ILK). When ILK is knocked-down, β1-integrin expression is significantly lowered following transfection of primary brain microvessel endothelial cells and it is associated with a decrease in claudin-5 expression, and a small change in F-actin. ILK is vital for EC survival, vascular development, and cell integrin–matrix interactions in mice. Yet, little is known about the role(s) of ILK in tight junction protein expression or permeability in the CNS in inflammatory processes (Izawa et al., 2018). In a study that aimed to discover the mechanism of function of TGF-β signaling in dermal lymphatic endothelial cells (LECs) epithelial–mesenchymal transition (EMT), it was found that TGF-β augmented the expression level of ILK Human lens epithelial cells (HLEC-h3), stimulated migration of HLEC-h3 cells, increased the level of E-cadherin protein, and lowered the expression of α-SMA protein, playing an important role in fibrogenesis. However, treatment with ILK siRNA, ILK inhibitor, and NF-κB inhibitor counteracted the effects of TGF-β on HLEC-h3 cells (Zhang and Huang, 2018). In our study, ILK was a target of the lncRNA RP11-732A19.2, differentially expressed in our VALs group which could indicate a possible mechanism by which γ-valerolactones inhibit the inflammatory process of TNF-α. Avoiding neuronal damage and neuronal death can have a significant clinical benefit. Flavonoids can be important compounds for the progress of novel therapeutic agents that could help in the effective treating of neurodegenerative diseases. Regular intake of flavonoids was observed to be associated with a reduced risk of neurodegenerative diseases (Solanki et al., 2015). The molecular targets identified in our study will supply the basis for the development of future therapeutic targets.

## Data Availability Statement

The datasets presented in this study can be found in online repositories. The names of the repository/repositories and accession number(s) can be found in the article/Supplementary Material.

## Author Contributions

KC, SN, and DM performed bioinformatic analyses and wrote the manuscript. SN and DM performed microarray analysis. DM designed the research and had primary responsibility for final content. KC, SN, JR, AV, CM, and DM participated in the interpretation of the data. HS contributed to the design of the experiment with flavanol metabolites. All authors read and approved the final manuscript.

## Conflict of Interest

DM initiated the study that was partially funded through an unrestricted research grant that he received from Mars Inc. Mars, Inc. also provided the epicatechin metabolites used in this study. HS was employed by Mars Inc., a company engaged in flavanol research and flavanol-related commercial activities. The remaining authors declare that the research was conducted in the absence of any commercial or financial relationships that could be construed as a potential conflict of interest. The reviewer CS declared a past co-authorship with one of the authors DM to the handling editor.

## Footnotes

1 www.uniprot.org/proteomes/UP000005640

2 http://thegpm.org.crap

3 https://github.com/dphansti/Sushi

4 https://github.com/cran/VennDiagram/blob/master/man/VennDiagram-package.Rd

5 https://biit.cs.ut.ee/clustvis/

6 http://rtools.cbrc.jp/LncRRIsearch

7 http://rtools.cbrc.jp/cgi-bin/RNARNA/index.pl

8 https://string-db.org/

9 https://amp.pharm.mssm.edu/Enrichr/

10 https://www.grnpedia.org/trrust/

11 https://pubchem.ncbi.nlm.nih.gov

12 https://bio-hpc.ucam.edu/Achilles

13 https://genetrail2.bioinf.uni-sb.de/

14 www.cytoscape.org/

15 https://github.com/mrdwab/splitstackshape

16 https://github.com/Rdatatable/data.table

17 http://dplyr.tidyverse.org,https://github.com/tidyverse/dplyr

18 http://stringr.tidyverse.org

19 https://github.com/tidyverse/stringr

20 http://apps.cytoscape.org/apps/networkanalyzer

## Supplementary Material

Supplementary Figure 1 Chemical structures of microbiota epicatechin metabolites.

Supplementary Figure 2 miRNA-enriched pathway interactions network. Pathway interactions network built in Cytoscape software to show the connections (edges) between enriched pathways (nodes) by miRNAs differentially expressed.

Supplementary Figure 3 Venn diagrams of mRNAs, miRNAs targets, lncRNAs targets, proteins and pathways related in human brain microvascular endothelial cells- γ-valerolactone treatment cell. **(A)** Venn diagram indicates the relationships between the number of mRNAs, miRNAs targets, lncRNA targets, and proteins differentially expressed. **(B)** Venn diagram indicates the relationships between the number of enriched pathways-mRNAs, miRNAs targets, lncRNA targets and proteins differentially expressed.

Supplementary Table 1 Effect of the γ-Valerolactones treatment on the expression of protein-coding genes in human brain microvascular endothelial cells: mRNAs differentially expressed.

Supplementary Table 2 Effect of the γ-Valerolactones treatment on the expression of miRNAs in human brain microvascular endothelial cells: miRNAs differentially expressed.

Supplementary Table 3 Effect of the γ-Valerolactones treatment on the expression of lncRNAs in human brain microvascular endothelial cells: lncRNAs differentially expressed.

Supplementary Table 4 Effect of the γ-Valerolactones treatment on the expression of proteins in HBMEC cells: proteins differentially expressed.
