# Supplementary material for: Integrated Multi-Omic Analyses of the Genomic Modifications by Gut Microbiome-Derived Metabolites of Epicatechin, 5-(4′-Hydroxyphenyl)-γ-Valerolactone, in TNFalpha-Stimulated Primary Human Brain Microvascular Endothelial Cells
Source: Front Neurosci. 2021 Mar 26;15:622640. doi: 10.3389/fnins.2021.622640 (PMC8033932; doi:10.3389/fnins.2021.622640)
Supplement: Supplementary Figure 1 — Chemical structures of microbiota epicatechin metabolites. [file Data_Sheet_1.PDF]

Supplemental Figure 1: Chemical structures of microbiota epicatechin metabolites

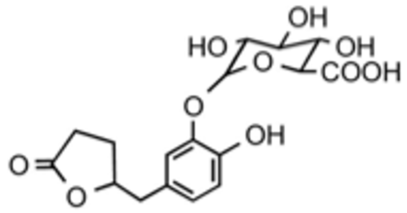

5-(4'-Hydroxyphenyl)-γ-valerolactone-3'-O-glucuronide

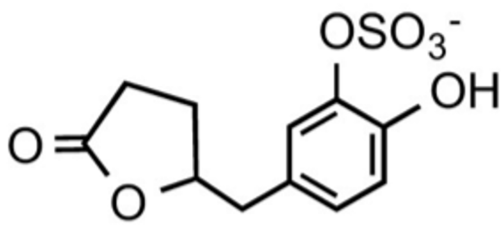

5-(4′-Hydroxyphenyl)-γ-valerolactone-3′-sulfate

Supplemental Figure 2. miRNA-enriched pathway interactions network.

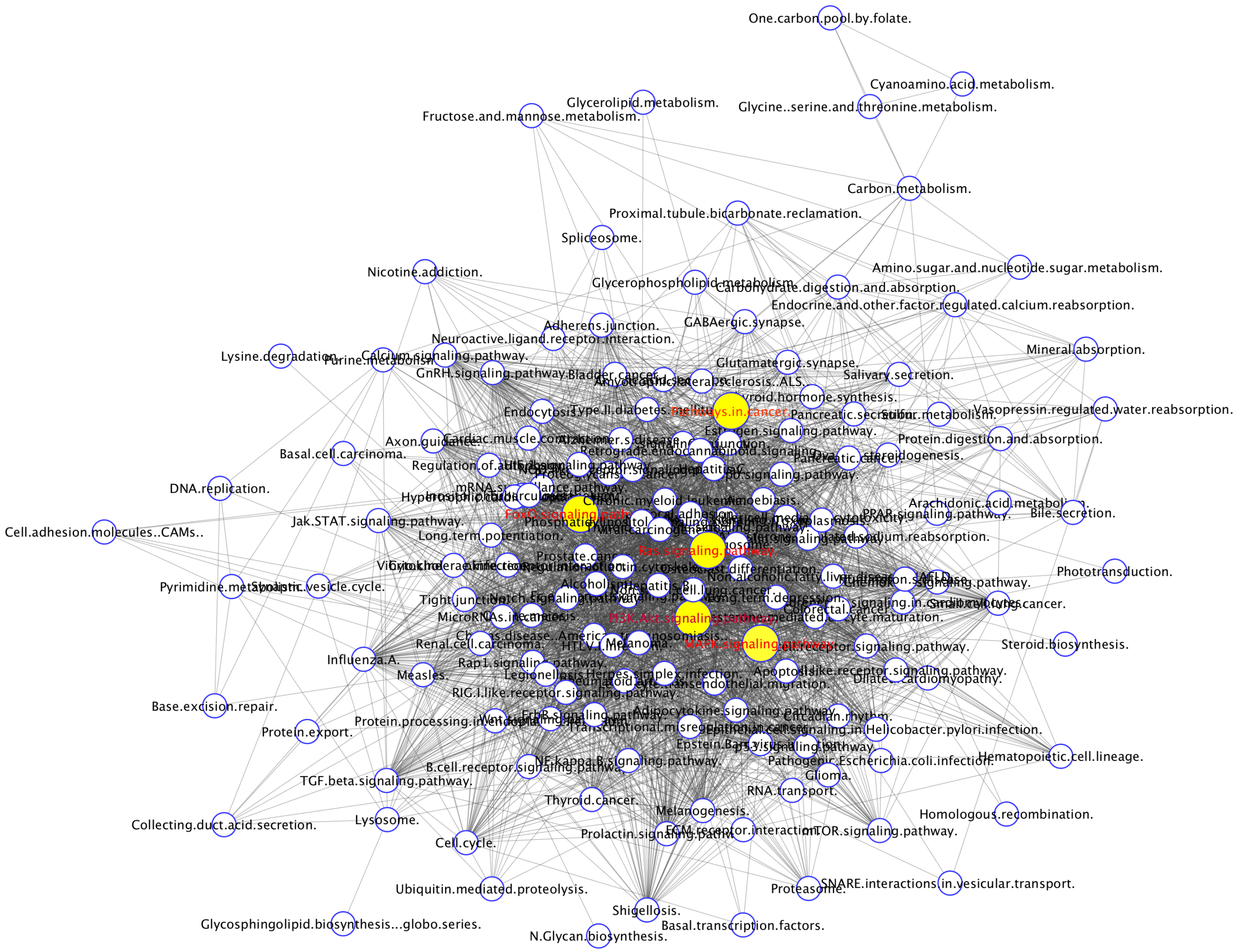

Supplemental Figure 3. Venn diagrams of mRNAs, miRNAs targets, lncRNAs targets, proteins and pathways related in HBMEC-valerolactone treatment cell.

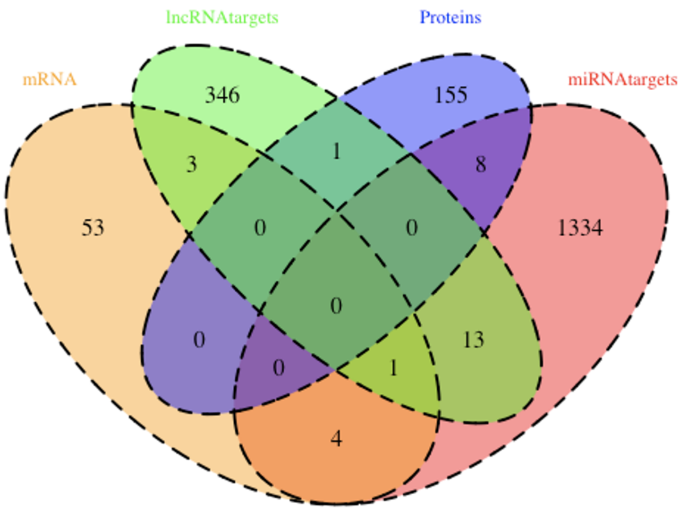

A) Venn diagram of genes

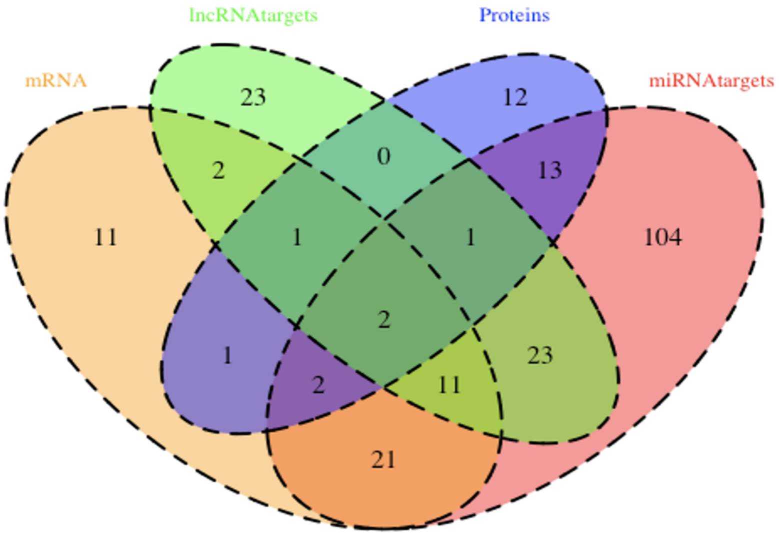

B)Venn diagram of pathways
